# Mutational spectrum and prognosis in NRAS-mutated acute myeloid leukemia

**DOI:** 10.1038/s41598-020-69194-6

**Published:** 2020-07-22

**Authors:** Shujuan Wang, Zhenzhen Wu, Tao Li, Yafei Li, Weiqiong Wang, Qianqian Hao, Xinsheng Xie, Dingming Wan, Zhongxing Jiang, Chong Wang, Yanfang Liu

**Affiliations:** Department of Hematology, The First Affiliated Hospital of Zhengzhou University, No. 1 Jianshe East Road, Erqi District, Zhengzhou, 450052 China

**Keywords:** Cancer, Genetics

## Abstract

The mutational spectrum and prognostic factors of *NRAS-*mutated (*NRAS*^mut^) acute myeloid leukemia (AML) are largely unknown. We performed next-generation sequencing (NGS) in 1,149 cases of de novo AML and discovered 152 *NRAS*^mut^ AML (13%). Of the 152 *NRAS*^mut^ AML, 89% had at least one companion mutated gene. DNA methylation-related genes confer up to 62% incidence. *TET2* had the highest mutation frequency (51%), followed by *ASXL1* (17%), *NPM1* (14%), *CEBPA* (13%), *DNMT3A* (13%), *FLT3-ITD* (11%), *KIT* (11%), *IDH2* (9%), *RUNX1* (8%), *U2AF1* (7%) and *SF3B1*(5%). Multivariate analysis suggested that age ≥ 60 years and mutations in *U2AF1* were independent factors related to failure to achieve complete remission after induction therapy. Age ≥ 60 years, non-M3 types and *U2AF1* mutations were independent prognostic factors for poor overall survival. Age ≥ 60 years, non-M3 types and higher risk group were independent prognostic factors for poor event-free survival (EFS) while allogenic hematopoietic stem cell transplantation was an independent prognostic factor for good EFS. Our study provided new insights into the mutational spectrum and prognostic factors of *NRAS*^mut^ AML.

## Introduction

Over the last two decades, our understanding of the molecular heterogeneity of acute myeloid leukemia (AML) has made significant advances through genomic discovery studies utilizing microarray and next-generation sequencing (NGS)- based “-omics ” technologies^1^. *RAS* oncogenes play important roles in diverse cellular events such as cell cycle, cell differentiation and survival^2^. *RAS* malfunction is strongly related to tumorigenesis and thus regarded as an important therapeutic target^3^. Mutations in the *RAS* genes (including *KRAS*, *NRAS* and *HRAS*) are discovered in 30% of all tumors^4^. *KRAS* is the most frequently mutated gene in cancers found in pancreatic (90%), colon (45%) and lung (35%), while *NRAS* mutations are more common in AML (10%)^4,5^.

Until now, the prognostic value of *NRAS* mutations in AML remains inconclusive. Recently, an integrated meta-analysis revealed that *NRAS* mutations did not influence the overall survival for adults with AML^6^. However, most of these reports evaluated *NRAS* in a binary fashion. The significance of variant allele frequency (VAF) of *NRAS* mutation at diagnosis, and the effect of companion gene mutations (co-mutations) in *NRAS*-mutated (*NRAS*^mut^) AML are warranted for extensive evaluation. In this study, we examined patient outcomes in a series of *NRAS*^mut^ de novo AML cases in terms of co-mutations and the NRAS VAF at diagnosis.

## Subjects and methods

### Patients

NGS was performed in 1,149 cases of de novo AML at the First Affiliated Hospital of Zhengzhou University between June 2016 and September 2019. One hundred and fifty-two cases with *NRAS*^mut^ AML were screened out and enrolled in the study. The diagnosis and classification of AML were based on the WHO 2016 edition of classification of myeloid neoplasms and acute leukemia^7^. Patients were divided into good, intermediate and poor risk group according to “Chinese Guidelines for Diagnosis and Treatment of Adult Acute Myeloid Leukemia (Not APL) (2017)”^8,9^. The study was approved by the Ethics Committee of the First Affiliated Hospital of Zhengzhou University. Informed consent was obtained from all patients or their statutory guardian following the Declaration of Helsinki.

### Treatment protocols

For M3 patients, all-trans retinoic acid and arsenic trioxide-based chemotherapy was given for the induction and consolidation therapy. Non-M3 patients received induction chemotherapy regimens include DA, IA, and MA regimens: a standard dose of cytarabine (Ara-C; 100 mg/m2/ day for 7 days) combined with daunorubicin (45 mg/m^2^/day for 3 days) or idarubicin (10 mg/m^2^/day for 3 days) or mitoxantrone (10 mg/m^2^/day for 3 days). After remission, young patients were consolidated with cytarabine (2–3 g/m^2^, once every 12 h for 3 days) based chemotherapy. For elderly patients, the chemotherapy consolidation was decided by the physicians in an individualized manner. A total of 24 patients underwent allogenic hematopoietic stem cell transplantation (allo-HSCT). Therapy recommendation was based on risk stratification and the results of minimal residual disease testing after 1–2 cycles of consolidation chemotherapy. The real treatment selection was based on both the physician’s recommendation and the patient’s preference. The last follow-up for surviving patients was conducted in December 2019.

### Cytogenetics and fusion genes analysis

Cytogenetic analyses were performed by G-banding analysis according to the International System for Human Cytogenetic Nomenclature. Forty-three fusion genes including *MLL-(AF4, AF6, AF9, AF10, AF17, AF1q, AF1p, AFX, ELL, SEPT6, ENL), NUP98-(HoxA9, HoxC11, HoxA11, HoxD13, HoxA13, PMX1), (NPM, FIP1L1, PML, PRKAR1A, STAT5b, NUMA1, PLZF)-RARα, (ETV6, FIP1L1)-PDGFRA, AML1-(ETO, MTG16, MDS1/EVI1), TEL-(JAK2, AML1, ABL), NPM-(ALK, MLF), (DEK, SET)-CAN, SIL-TAL1, E2A-HLF, TEL-PDGFRB, TLS-ERG, CBFβ-MYH11, E2A-PBX1* and *BCR-ABL* were detected with real-time PCR (RT-PCR) using Multiplex RT-PCR Fusion Gene Kits (Rightongene, Shanghai, China).

### Next generation sequencing

We sequenced the mutational hotpots or whole coding regions of 22 genes (including *FLT3*, *NPM1*, *KIT*, *CEBPA*, *DNMT3A*, *IDH1*, *IDH2*, *TET2*, *EZH2*, RUNX1, *ASXL1*, *PHF6*, *TP53*, *SF3B1*, *SRSF2*, *U2AF1*, *ZRSR2*, *NRAS*, *CBL*, *SETBP1*, *ETV6*, and *JAK2*) with the standard NGS technology. The detection was based on a Illumina MiSeq System (Illumina, San Diego, CA) high-throughput sequencing platform by using a Rightongene AML/MDS/MPN Sequencing Panel (Rightongene, Shanghai, China). Details of the variant calling, filtering, and annotation are described in our recently published reports^10^.

### Statistical analysis

Analyses were performed using SPSS software version 20.0 (Chicago, IL, USA) and Graphpad Prism™ 5.01 (San Diego, California, USA). Differences across groups were compared using the *Pearson Chi-square analysis* or *Fisher exact test* for categorical variables, and *Mann–Whitney U* test for continuous variables. Overall survival (OS) is defined as the time from diagnosis to death or the time of the last follow-up. Event-free survival (EFS) is defined as the time from diagnosis to relapse, death, or the time of the last follow-up. Survival analysis was estimated by *Kaplan–Meier* method and compared by the *log-rank* test. Multivariable analysis including variables with *P* < 0.10 in univariate analysis were performed for complete remission (CR), OS and EFS. *P* < 0.05 was considered to indicate statistical significance.

## Results

### Clinical and biological characteristics of ***NRAS***^mut^ AML

In the total cohort, *NRAS* mutations were found in 13% (152 of 1,149) cases. As shown in Table 1, median age was 44 (range 14–78) years, with 25 cases (16%) older than 60 years. Half of the cases were men. Twelve cases (8%) were M3 and 140 cases were non-M3 AML. The median white blood cell (WBC) count at diagnosis was 31 × 10^9^/L, and in 27 cases (18%) it was ≥ 100 × 10^9^/L. Forty-five cases (30%) had a bone marrow blast percentage of more than 80%. Forty-one cases (27%) was good-risk AML, 64 (42%) was intermediate-risk AML and 47 (31%) was poor-risk AML. Twenty-four cases (16%) received allogenic hematopoietic stem cell transplantation (allo-HSCT). Thirty-six cases failed to achieve CR after induction therapy and 61 cases died at the end of follow up. Thirty-five cases (16%) had more than two other recurrent genetic mutations. Forty-three fusion genes were detected in 135 cases and 16 cases were AML1-ETO positive; 15 cases were MYH11-CBFβ positive and 7 cases were MLL positive.

**Table 1:** Clinical and molecular characteristics of *NRAS*^mut^ AML.

Characteristics	Median (interquartile range) or N (%)
Gender (male)	76 (50%)
Age (years)	44 (30–55)
Age ≥ 60 years	25 (16%)
M3	12 (8%)
***NRAS type***	
G12/G13	120 (79%)
Q61	26 (17%)
Mix	5 (3%)
* NRAS* VAF (%)	15 (6–33)
* NRAS* VAF (≥ 15%)	76(50%)
WBC counts (× 10^9^/L)	31 (9–75)
WBC counts (≥ 100 × 10^9^/L)	27 (18%)
HGB counts (g/L)	75 (63–89)
HGB counts (≥ 110 g/L)	15 (10%)
PLT counts (× 10^9^/L)	31 (15–71)
PLT counts (≥ 100 × 10^9^/L)	20 (13%)
BM blasts (%)	59 (36–85)
BM blasts (≥ 80%)	45 (30%)
**Fusion genes (N = 135)**	
* AML1-ETO*	16 (12%)
* MYH11-CBFβ*	15 (11%)
* MLL*	7 (5)
* BCR-ABL*	2 (1)
*SET-CAN*	1 (1)
* NUP98*	1 (1)
* DEK-CAN*	1 (1)
* AML-EVI1*	1 (1)
* AML-MTG1*	1 (1)
** Risk group**	
Good	41 (27%)
Intermediate	64 (42%)
Poor	47 (31%)
Number of co-mutations (≥ 3)	35 (23%)
Allo-HSCT	24 (16%)
Non-CR	36 (24%)
Death	61 (40%)

*VAF* Variant allele frequency, *WBC* white blood cell, *HGB* hemoglobin, *PLT* platelets, *BM* bone marrow, *allo-HSCT* allogenic hematopoietic stem cell transplantation, *CR* complete remission.

**Figure 1:**
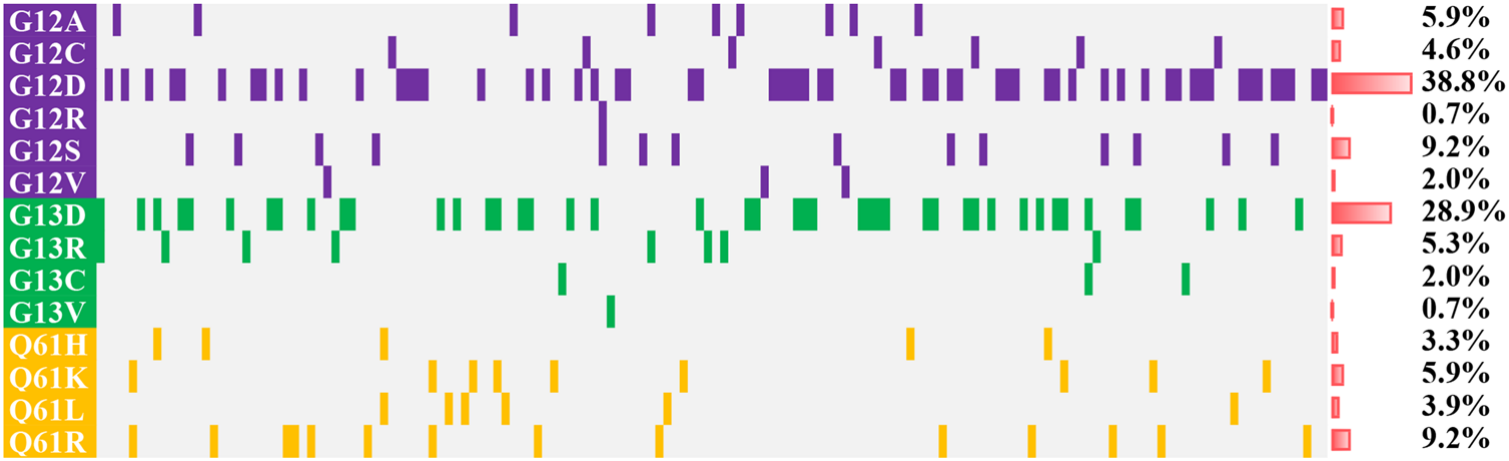
*NRAS* mutations at codon 12, 13 and 61 of 152 de novo AML patients. Distribution and frequencies are given for *NRAS* mutations at codon 12, 13 and 61. The boxes in one column represent single patient cases. Mutations were color coded by mutation type. The histogram on the right showed the frequency distribution of all aberrations.

Most *NRAS* mutations (88 of 152; 57.9%) were found at codon 12. Mutations at codon 13 were found in 54 (35.5%) of 152 cases. The most frequent changes were from glycine to asparagine (codon 12: G12D, 59 of 152, 38.8%; codon 13: G13D, 44 of 152, 28.9%; Fig. 1). Mutations at codon 61 were detected in 31 (20.4%) of 152 cases, mostly from glycine to arginine (Q61R, 14 of 152, 9.2%; Fig. 1). It is worth noting that NRAS mutations at codons 146 which was a noncanonical N-RAS mutation were detected in one case. *NRAS* mutation types were divided into G12/13, Q61 and mix (G12/13 and Q61), with G12/13 accounting for 79% of the cases (Table 1). The median VAF of *NRAS* was 15% (range 1–59%).

### Companion gene mutations in *NRAS*^mut^ AML

**Figure 2:**
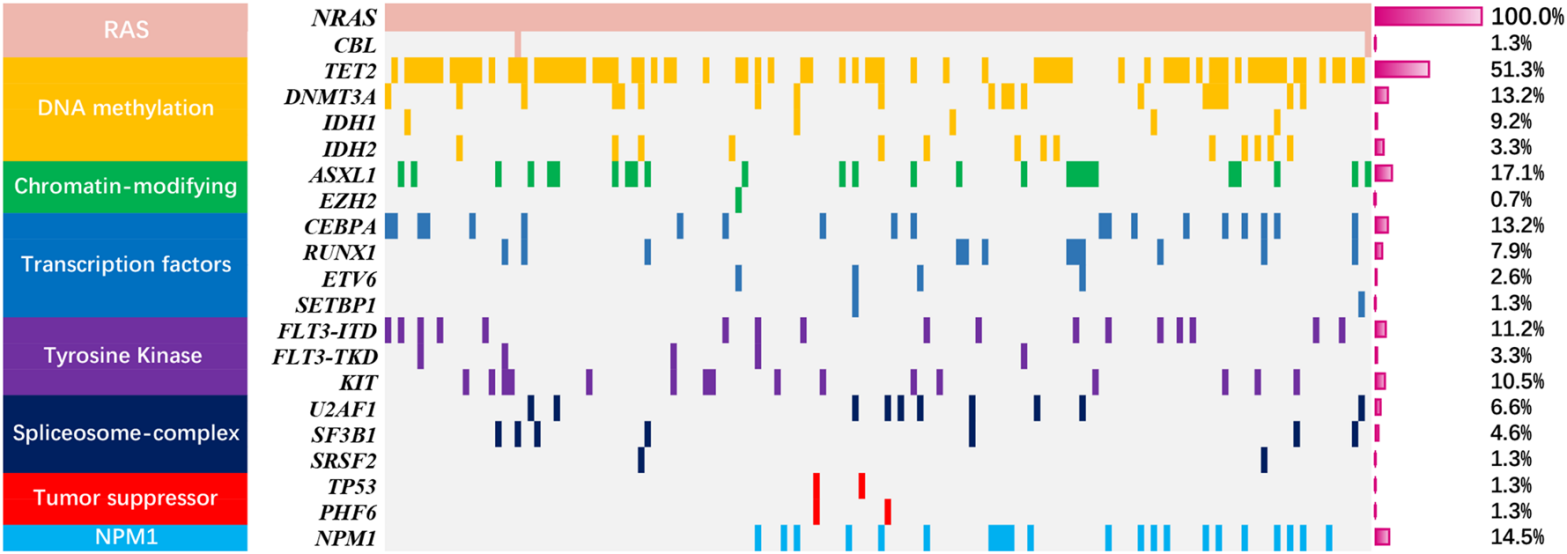
Mutational landscape of 152 *NRAS*^mut^ AML patients. The landscape showed all genetic aberrations for each patient. The boxes in one column represent single patient cases. Mutations were color coded by mutation type. The histogram on the right showed the frequency distribution of all aberrations.

One hundred and thirty-five cases (89%) had at least one co-mutation besides *NRAS*. Fifty-four cases had one co-mutation, 46 cases with 2, 22 cases with 3, 10 cases with 4 and 3 cases with 5. As shown in Fig. 2, *TET2* had the highest mutation frequency (51%), followed by *ASXL1* (17%), *NPM1* (14%), *CEBPA* (13%), *DNMT3A* (13%), *FLT3-ITD* (11%), *KIT* (11%), *IDH2* (9%), *RUNX1* (8%), *U2AF1* (7%) and *SF3B1*(5%). Other mutated genes (including *CBL*, *IDH2*, *EZH2*, *ETV6*, *SETBP1*, *FLT3-TKD*, *SRSF2*, *TP53*, *PHF6*) are less than 5% in *NRAS*^mut^ AML; *JAK2* and *ZRSR2* mutations are absent in *NRAS*^mut^ AML. These gene mutations are further classified into functional groups as previously described: *RAS* pathway (100%)-*NRAS* and *CBL*; DNA methylation (62%)—*TET2*, *DNMT3A* and *IDH1/2*; chromatin modifying (18%)—*ASXL1*, *EZH2*; transcription factors (22%)-*CEBPA*, *RUNX1*, *ETV6* and *SETBP1*; Tyrosine kinase (7%)—*FLT3-ITD/TKD*, *KIT* and *JAK2*; Spliceosome complex (12%)- *U2AF1*, *SF3B1*, *SRSF2* and *ZRSR2*; Tumor suppressor (2%)-*TP53* and *PHF6*; *NPM1* gene (14%)-*NPM1*.

### Response to induction therapy

One hundred and sixteen cases achieved CR after 1–3 cycles of induction therapy while 36 cases didn’t achieve CR. We validated the prognostic value of clinical variables and other genetic mutations in response to induction therapy. Comparison analysis was conducted considering variables such as gender (female *vs*. male), age (≥ 60 *vs*. < 60 years), AML types (Non-M3 *vs*. M3), *NRAS* type (mix *vs.* Q61 vs. G12/G13), *NRAS* VAF (≥ 15% *vs.* < 15%), WBC counts (≥ 100 *vs*. < 100 × 10^9^/L), HGB counts (≥ 110 *vs*. < 110 g/L), PLT counts (≥ 100 *vs*. < 100 × 10^9^/L), bone marrow blasts (≥ 80% *vs*. < 80%), peripheral blood blasts (≥ 20% *vs*. < 20%), number of co-mutations (≥ 3 *vs*. < 3), allo-HSCT (yes *vs*. no), risk group (high *vs*. inter *vs*. low -risk), *ETO* (positive *vs*. negative), *MYH11-CBFβ* (positive *vs*. negative), and the mutation status of other common AML co-mutations. In univariate analysis, it was shown that age ≥ 60 years, higher risk group, *U2AF1* mutations and *SF3B1* mutations were associated with lower CR rate (Table 2). While other factors were not associated with the induction outcome of the *NRAS*^mut^ AML patients (Table 2). In multivariate analysis, it was shown that age ≥ 60 years and *U2AF1* mutations were independent prognostic factors for response to induction therapy (Table 2).

**Table 2:** Univariate analysis and multivariate analysis of response to induction therapy in NRAS^mut^ AML.

Variables	Univariate analysis		Multivariate analysis	
	OR (95% CI)	*P* value	OR (95% CI)	*P* value
Gender (female)	1.80 (0.84–3.87)	0.130		
Age ≥ 60 years	0.08 (0.03–0.22)	0.000	0.06 (0.02–0.20)	0.000
Non-M3	0.27 (0.03–2.19)	0.221		
	0.54 (0.27–1.07)	0.078	0.46 (0.18–1.17)	0.105
*NRAS* VAF ≥ 15%	0.75 (0.35–1.58)	0.446		
WBC ≥ 100 × 10^9^/L	2.87 (0.81–10.16)	0.102		
HGB ≥ 110 g/L	1.27 (0.34–4.77)	0.724		
PLT ≥ 100 × 10^9^/L	0.69 (0.24–1.94)	0.478		
BM blasts ≥ 80%	1.64 (0.68–3.94)	0.270		
*ETO*	2.36 (0.51–10.99)	0.274		
*MYH11-CBFβ*	1.28 (0.34–4.83)	0.721		
Risk group		0.000		0.052
Good vs. poor	5.82 (1.94–17.44)	0.002	1.53 (0.36–6.55)	0.568
Inter vs. poor	4.36 (1.78–10.58)	0.001	4.89 (1.33–17.97)	0.017
Co-mutations ≥ 3	0.87 (0.36–2.07)	0.748		
*TET2*	0.69 (0.32–1.47)	0.336		
*ASXL1*	0.42 (0.17–1.02)	0.056	1.50 (0.36–6.29)	0.577
*NPM1*	7.74 (1.00–59.69)	0.050	4.20 (0.37–47.21)	0.245
*FLT3-ITD*	0.72 (0.23–2.19)	0.557		
*CEBPA*	6.86 (0.89–53.13)	0.065	10.05 (0.56–181.60)	0.118
*DNMT3A*	3.12 (0.69–14.16)	0.140		
*KIT*	5.20 (0.66–40.80)	0.117		
*IDH2*	0.76 (0.22–2.57)	0.653		
*RUNX1*	0.93 (0.24–3.62)	0.911		
*U2AF1*	0.03 (0.00–0.22)	0.001	0.03 (0.00–0.30)	0.004
*SF3B1*	0.11 (0.02–0.59)	0.010	0.28 (0.03–2.72)	0.271

*OR* Odds ratio, VAF variant allele frequency, WBC white blood cell, HGB hemoglobin, PLT platelets, BM bone marrow.

### Comparison of OS and EFS between different clinical and molecular characteristic groups

**Figure 3:**
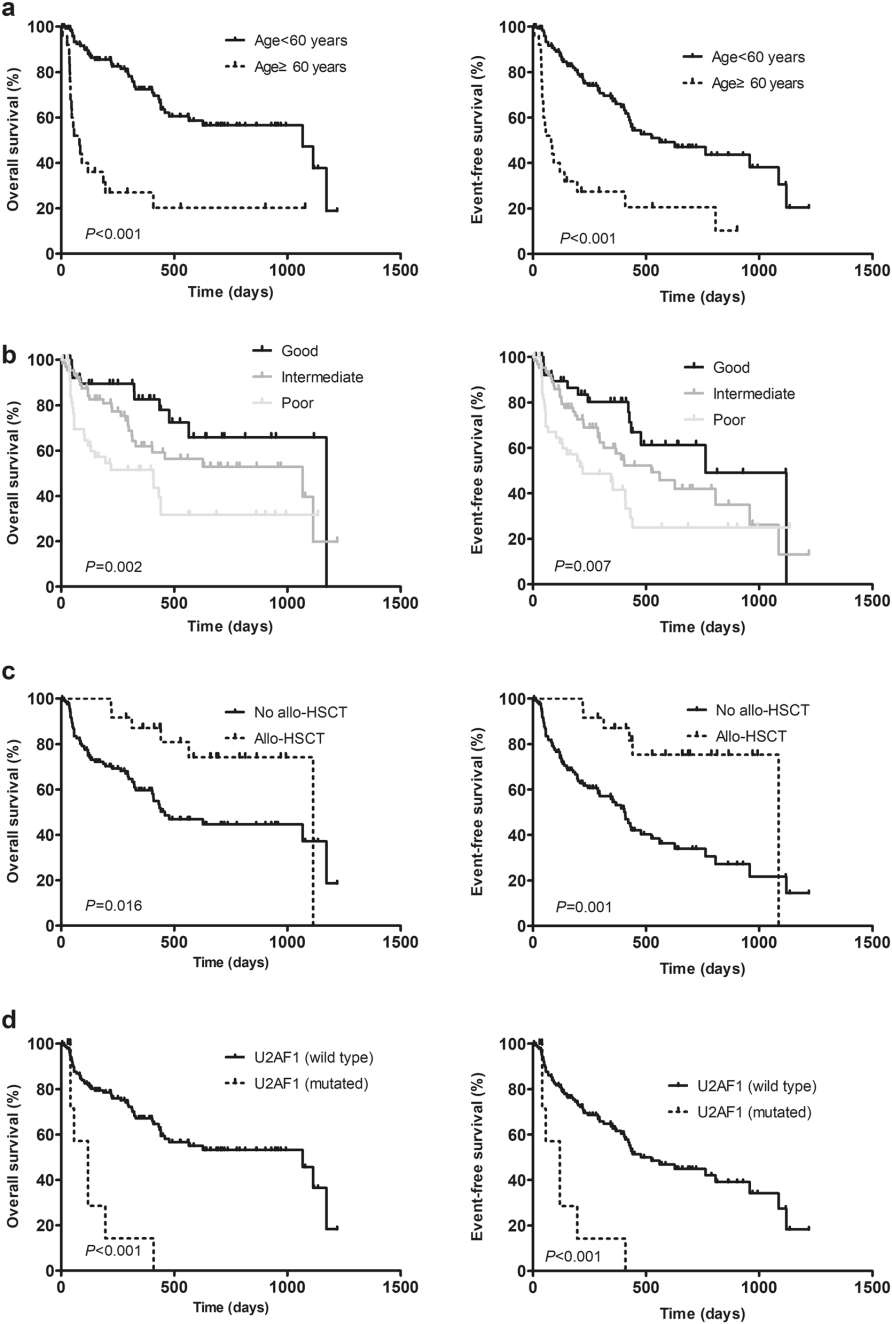
Kaplan–Meier estimate of overall survival (OS) and event-free survival (EFS) in 152 *NRAS*^mut^ AML. OS and EFS were compared in (**a**) patients older than 60 years and patients younger than 60 years (**b**) different risk groups (**c**) patients who accepted allo-HSCT or not (d) patients with *U2AF1* mutations or not.

Comparison analysis of EFS and OS was conducted considering variables such as different gender (female vs. male), age (≥ 60 *vs*. < 60 years), AML types (Non-M3 *vs*. M3), *NRAS* type (mix *vs*. Q61 *vs*. G12/G13), *NRAS* VAF (≥ 15% *vs*. < 15%), WBC counts (≥ 100 *vs*. < 100 × 10^9^/L), HGB counts (≥ 110 *vs*. < 110 g/L), PLT counts (≥ 100 *vs*. < 100 × 10^9^/L), bone marrow blasts (≥ 80% *vs*. < 80%), peripheral blood blasts (≥ 20% *vs*. < 20%), number of co-mutations (≥ 3 *vs*. < 3), allo-HSCT (yes *vs*.no), risk group (high *vs*. inter *vs*. low -risk), ETO (positive *vs*. negative), MYH11-CBFβ (positive *vs*. negative), and the mutation status of other common AML co-mutations. The median follow-up time was 294 (5–1,219) days. As shown in Table 3, older cases (age ≥ 60 years) had shorter OS and EFS (*P* = 0.000, *P* = 0.000, respectively; Fig. 3a). M3 cases had longer OS and EFS (*P* = 0.030, *P* = 0.008, respectively). Higher risk group was associated worse OS and EFS (*P* = 0.002, *P* = 0.007, respectively; Fig. 3b). Cases who accepted allo-HSCT had longer OS and EFS (*P* = 0.016, *P* = 0.001, respectively; Fig. 3c). Presence of U2AF1 was associated with shorter OS and EFS (*P* = 0.000, *P* = 0.000, respectively; Fig. 3d). Presence of RUNX1 and SF3B1 was associated with shorter OS (*P* = 0.014, *P* = 0.032, respectively). Number of co-mutations ≥ 3 and presence of IDH2 was associated with shorter EFS (*P* = 0.025, *P* = 0.043, respectively). However, both NRAS type and NRAS VAF had no effect on EFS and OS.

**Table 3:** Comparison of EFS and OS between different clinical and molecular characteristic groups in NRAS^mut^ AML.

Variables	OS		EFS	
	*χ2*	*P *value	*χ2*	*P *value
Gender (female vs. male)	0.000	0.985	0.020	0.888
Age (≥ 60 vs. < 60 years)	36.959	0.000	30.844	0.000
AML types (non-M3 vs. M3)	4.709	0.030	6.949	0.008
*NRAS* type (mix vs. Q61 vs. G12/G13)	2.133	0.348	2.049	0.359
*NRAS* VAF (≥ 15% vs. < 15%)	0.071	0.790	0.000	0.991
WBC counts (≥ 100 vs. < 100 × 10^9^/L)	0.343	0.558	1.558	0.212
HGB counts (≥ 110 vs. < 110 g/L)	0.148	0.700	0.032	0.857
PLT counts (≥ 100 vs. < 100 × 10^9^/L)	1.197	0.274	1.992	0.158
BM blasts (≥ 80% vs. < 80%)	0.567	0.451	0.683	0.408
PB blasts (≥ 20% vs. < 20%)	0.872	0.350	0.430	0.512
Risk group (high vs. inter vs. low -risk)	12.549	0.002	10.029	0.007
Allo-HSCT (yes vs.no)	5.777	0.016	10.808	0.001
*ETO* (positive vs. negative)	1.753	0.185	1.337	0.248
*MYH11-CBFβ* (positive vs. negative)	0.300	0.584	0.352	0.553
Number of co-mutations (≥ 3 vs. < 3)	2.433	0.118	5.055	0.025
*TET2* (mutated vs. wild type)	0.325	0.569	0.593	0.441
*ASXL1*(mutated vs. wild type)	3.625	0.057	1.048	0.306
*NPM1* (mutated vs. wild type)	1.009	0.315	0.280	0.596
*CEBPA* (mutated vs. wild type)	1.982	0.159	0.187	0.666
*DNMT3A* (mutated vs. wild type)	0.233	0.629	0.022	0.881
*FLT3-ITD* (positive vs. negative)	0.220	0.639	1.142	0.285
*KIT* (mutated vs. wild type)	1.804	0.179	0.631	0.427
*IDH2* (mutated vs. wild type)	0.090	0.764	4.095	0.043
*RUNX1* (mutated vs. wild type)	6.075	0.014	3.407	0.065
*U2AF1* (mutated vs. wild type)	18.556	0.000	15.464	0.000
*SF3B1* (mutated vs. wild type)	4.578	0.032	2.511	0.113

*VAF* Variant allele frequency, *WBC* white blood cell, *HGB* hemoglobin, *PLT* platelets, *BM* bone marrow, *PB* peripheral blood, *allo-HSCT* allogenic hematopoietic stem cell transplantation.

### Evaluation of possible prognostic factors

Multivariate analysis of factors related to OS included age (≥ 60 *vs*. < 60 years), AML types (non-M3 *vs*. M3), risk group (high *vs*. inter *vs*. low-risk), the time-dependent variable for allo-HSCT (yes *vs*.no), *ASXL1* (mutated vs. wild type), *RUNX1* (mutated vs. wild type), *U2AF1* (mutated *vs*. wild type) and *SF3B1* (mutated *vs*. wild type). As shown in Table 4, independent prognostic factors for poor OS included age ≥ 60 years, non-M3 types and *U2AF1* mutations.

**Table 4:** Multivariate analysis for OS and EFS in NRAS^mut^ AML.

Variables	OS		EFS	
	HR (95% CI)	*P *value	HR (95% CI)	*P *value
Age ≥ 60 years	2.85 (1.53–5.33)	0.001	1.99 (1.09–3.62)	0.025
Non-M3	7.94 (1.06–59.65)	0.044	11.22 (1.50–84.11)	0.019
Risk group		0.055		0.037
Good vs. poor	0.40 (0.18–0.90)	0.026	0.38 (0.18–0.81)	0.012
Inter vs. poor	0.57 (0.31–1.04)	0.065	0.61(0.35–1.08)	0.088
Allo-HSCT*	0.89 (0.76–1.04)	0.134	0.81 (0.70–0.95)	0.009
Co-mutations ≥ 3			1.22 (0.66–2.24)	0.529
*ASXL1*	1.24 (0.61–2.50)	0.554		
*IDH2*			1.37 (0.65–2.89)	0.411
*RUNX1*	1.48 (0.59–3.76)	0.405	0.95 (0.36–2.46)	0.911
*U2AF1*	2.49 (1.05–5.89)	0.038	2.11 (0.90–4.98)	0.088
*SF3B1*	0.55 (0.17–1.73)	0.303		

*HR* Hazard ratio, *allo-HSCT* allogenic hematopoietic stem cell transplantation, allo-HSCT* the time-dependent variable for allo-HSCT (allo-HSCT × LN(T_)).

Multivariate analysis of factors related to EFS included age (≥ 60 *vs*. < 60 years), AML types (Non-M3 *vs*. M3), risk group (high *vs*. inter *vs*. low-risk), the time-dependent variable for allo-HSCT (yes *vs*.no), number of co-mutations (≥ 3 *vs*. < 3), *IDH2* (mutated *vs*. wild type), *RUNX1* (mutated *vs*. wild type) and *U2AF1* (mutated *vs*. wild type). As shown in Table 4, age ≥ 60 years, non-M3 types and higher risk group were independent prognostic factors for poor EFS while allo-HSCT was an independent prognostic factor for good EFS.

## Discussion

High frequencies of *NRAS* mutations had been seen in AML patients^11^, indicating its important function in the pathogenesis and progression of AML. Although the prognostic value of *NRAS* mutations in AML patients remains inconclusive^6,12^, several large cohort studies indicated that *NRAS* mutations in AML did not influence the prognosis of patients^11,13,14^. A recently published meta-analysis also suggested that *NRAS* oncogene mutations were not correlated with the prognosis of patients with AML^6^. However, given the prevalence of *NRAS* mutations in AML, there is urgently need to explore the clinical characteristics, companion gene mutations and possible prognostic factors of *NRAS*^mut^ AML patients to provide evidence for clinical stratified diagnosis and treatment.

Our data showed that *NRAS* mutations were found in 13% of cases, which is consistent with findings in other literature that showed a range of 9.7% to 13.9% *NRAS* mutations^11,14–16^. The median age of *NRAS*^mut^ AML cases was 44 years and the median WBC counts was 31 × 10^9^/L, which was consistent with a large cohort study in China in 2013^14^. In our study, most *NRAS* mutations were found at codon 12 and the most frequent change were from glycine to asparagine, which was supported by previous reports^11,17^. Interestingly, we found that some samples have two NRAS mutations such as Q61K + Q61R, which have been confirmed by Sanger sequencing. This situation is very rare, it is not ruled out that two mutations have occurred in the same gene, but it may also be caused by mutations in the same allele. Nearly 90% of the cases had at least one companion gene mutation, which suggests that the molecular mechanism of patients with *NRAS*^mut^ AML is complicated, and multiple molecular interactions may exist. However, previous studies often focused on comparing the difference between *NRAS*^mut^ and *NRAS* wild-type patients^11,14^, with little attention on the molecular mutation spectrum. We observed that mutations of DNA methylation-related genes occurred in 62% *NRAS*^mut^ AML, the most common of which is *TET2*. This indicated that DNA methylation may play an important role in the pathogenesis in *NRAS*^mut^ AML and provided a basis for demethylation treatment of *NRAS*^mut^ AML patients.

AML in older patients generally had poorer prognosis due to poorer baseline performance status, and co-morbidities^18^. In our cohort of *NRAS*^mut^ AML, age ≥ 60 years also had a negative impact on both response to induction therapy and survival. Allogeneic HSCT which was usually considered the cure for AML, also showed survival benefit in our study. Traditional risk stratification schemes based on genetics and molecular biology were still applicable in patients with *NRAS*^mut^ AML and could well predict patients’ prognosis. Mutation Gene VAF of *FLT3-ITD* or *NPM1* was reported to be significantly correlated with prognosis of AML^19,20^. However, we found that *NRAS* VAF had no correlation with either response to induction therapy or survival. *FLT3-ITD* was associated with increased risk of relapse while *NPM1*, *AML1-ETO*, *MYH11-CBFβ* were good prognostic factors^18^. In our study, however, recurrent genetic mutations including *FLT3-ITD*, *NPM1*, *DNMT3A*, *TET2* and *KIT* and fusion genes including *AML1-ETO* and *MYH11-CBFβ* were not associated with survival. The discrepancy may be related to possible interplay of mutated genes.

*U2AF1* belongs to mutations in splicing factor (SF) genes. Mutations in *U2AF1* is a poor prognostic indicator in myelodysplastic syndrome^21^. Recently many studies proved that mutations in *U2AF1* predict poor prognosis in patients with de novo AML^22–25^. Our study showed that *U2AF1* was also an independent poor prognostic factor for survival in *NRAS*^mut^ AML patients. In a large study of 664 AML patients conducted by the German AML Cooperative Group, mutations in *U2AF1* were one of the independent risk factors for achievement of CR1^26^. Similar to this result, in our study, 90% *U2AF1-*mutated AML patients failed to achieve complete remission.

The limitations to our study need to be acknowledged. First, our study was retrospective and susceptible to selection biases. Second, some gene mutations may not be detected due to the limitation of technique. Prognostic effects of some important gene mutations may be ignored. Third, whether these findings are restricted to *NRAS*^mut^ AML need to be further explored by parallel comparison with non *NRAS*^mut^ AML. Fourth, the small sample sizes of some subgroups resulted in relatively low statistical power and the univariate analyses were not adjusted for multiple comparisons which may result in false positive results, especially in small subgroups. Because of these limitations, our conclusion needs validation in a larger and prospective cohort.

In conclusion, our study provided new insights into the mutational spectrum and prognostic factors of *NRAS*^mut^ AML. These individuals companied with *U2AF1* mutations experienced poor responses to chemotherapy and the mechanisms need to further evaluate. More detailed mutational spectrum information and large prospective studies are needed in the future for better prognostication of *NRAS*^mut^ AML.

## References

[1] Bullinger L, Döhner K, Döhner H (2017). Genomics of acute myeloid leukemia diagnosis and pathways. J. Clin. Oncol..

[2] Renneville A (2008). Cooperating gene mutations in acute myeloid leukemia: a review of the literature. Leukemia.

[3] Cho H, Shin I, Ju E, Choi S, Hur W (2018). First SAR study for overriding NRAS mutant driven acute myeloid. Leukemia.

[4] Wilson CY, Tolias P (2016). Recent advances in cancer drug discovery targeting RAS. Drug Discov. Today.

[5] Nonami A (2015). Identification of novel therapeutic targets in acute leukemias with NRAS mutations using a pharmacologic approach. Blood.

[6] 6Liu, X. *et al.* RAS mutations in acute myeloid leukaemia patients: a review and meta-analysis. *Clin. Chim. Acta Int .J. Clin. Chem*. **489**, 254–260, 10.1016/j.cca.2018.08.040 (2019). 10.1016/j.cca.2018.08.040 30194935

[7] Arber DA (2016). The 2016 revision to the World Health Organization classification of myeloid neoplasms and acute leukemia. Blood.

[8] [Chinese guidelines for diagnosis and treatment of adult acute myeloid leukemia (not APL) (2017)]. *Zhonghua Xue Ye Xue Za Zhi* **38**, 177–182, 10.3760/cma.j.issn.0253-2727.2017.03.001 (2017). 10.3760/cma.j.issn.0253-2727.2017.03.001 PMC7348391 28395438

[9] Papaemmanuil E (2016). Genomic classification and prognosis in acute myeloid leukemia. N. Engl. J. Med..

[10] Yu J (2020). Gene mutational analysis by NGS and its clinical significance in patients with myelodysplastic syndrome and acute myeloid leukemia. Exp. Hematol. Oncol..

[11] Bacher U, Haferlach T, Schoch C, Kern W, Schnittger S (2006). Implications of NRAS mutations in AML: a study of 2502 patients. Blood.

[12] De Melo MB, Lorand-Metze I, Lima CS, Saad ST, Costa FF (1997). N-ras gene point mutations in Brazilian acute myelogenous leukemia patients correlate with a poor prognosis. Leukemia Lymphoma.

[13] Bowen DT (2005). RAS mutation in acute myeloid leukemia is associated with distinct cytogenetic subgroups but does not influence outcome in patients younger than 60 years. Blood.

[14] Yang X (2013). RAS mutation analysis in a large cohort of Chinese patients with acute myeloid leukemia. Clin. Biochem..

[15] Neubauer A (1994). Prognostic importance of mutations in the ras proto-oncogenes in de novo acute myeloid leukemia. Blood.

[16] Kiyoi H (1999). Prognostic implication of FLT3 and N-RAS gene mutations in acute myeloid leukemia. Blood.

[17] Reuter CWM (2014). Lack of noncanonical RAS mutations in cytogenetically normal acute myeloid leukemia. Ann. Hematol..

[18] Döhner H, Weisdorf DJ, Bloomfield CD (2015). Acute myeloid leukemia. N. Engl. J. Med..

[19] Patel SS (2018). High NPM1-mutant allele burden at diagnosis predicts unfavorable outcomes in de novo AML. Blood.

[20] Schlenk RF (2014). Differential impact of allelic ratio and insertion site in FLT3-ITD-positive AML with respect to allogeneic transplantation. Blood.

[21] Thol F (2012). Frequency and prognostic impact of mutations in SRSF2, U2AF1, and ZRSR2 in patients with myelodysplastic syndromes. Blood.

[22] Hou H-A (2016). Splicing factor mutations predict poor prognosis in patients with de novo acute myeloid leukemia. Oncotarget.

[23] Hamilton BK (2016). Impact of allogeneic hematopoietic cell transplant in patients with myeloid neoplasms carrying spliceosomal mutations. Am. J. Hematol..

[24] Saygin C (2018). Mutations in DNMT3A, U2AF1, and EZH2 identify intermediate-risk acute myeloid leukemia patients with poor outcome after CR1. Blood Cancer J..

[25] Ohgami RS (2015). Next-generation sequencing of acute myeloid leukemia identifies the significance of TP53, U2AF1, ASXL1, and TET2 mutations. Mod. Pathol..

[26] Metzeler KH (2016). Spectrum and prognostic relevance of driver gene mutations in acute myeloid leukemia. Blood.

